# Controversial Variable Node Selection-Based Adaptive Belief Propagation Decoding Algorithm Using Bit Flipping Check for JSCC Systems

**DOI:** 10.3390/e24030427

**Published:** 2022-03-19

**Authors:** Hao Wang, Wei Zhang, Yizhe Jing, Yanyan Chang, Yanyan Liu

**Affiliations:** 1 School of Microelectronics, Tianjin University, Tianjin 300072, China; ahao@tju.edu.cn (H.W.); tjuzhangwei@tju.edu.cn (W.Z.); jingyz1999@tju.edu.cn (Y.J.); yycsdut@tju.edu.cn (Y.C.); 2 College of Electronic Information and Optical Engineering, Nankai University, Tianjin 300071, China

**Keywords:** Reed–Solomon (RS) codes, adaptive belief propagation (ABP), JSCC system, joint coding matrix, bit flipping, coding gain

## Abstract

An end-to-end joint source–channel (JSC) encoding matrix and a JSC decoding scheme using the proposed bit flipping check (BFC) algorithm and controversial variable node selection-based adaptive belief propagation (CVNS-ABP) decoding algorithm are presented to improve the efficiency and reliability of the joint source–channel coding (JSCC) scheme based on double Reed–Solomon (RS) codes. The constructed coding matrix can realize source compression and channel coding of multiple sets of information data simultaneously, which significantly improves the coding efficiency. The proposed BFC algorithm uses channel soft information to select and flip the unreliable bits and then uses the redundancy of the source block to realize the error verification and error correction. The proposed CVNS-ABP algorithm reduces the influence of error bits on decoding by selecting error variable nodes (VNs) from controversial VNs and adding them to the sparsity of the parity-check matrix. In addition, the proposed JSC decoding scheme based on the BFC algorithm and CVNS-ABP algorithm can realize the connection of source and channel to improve the performance of JSC decoding. Simulation results show that the proposed BFC-based hard-decision decoding (BFC-HDD) algorithm (ζ = 1) and BFC-based low-complexity chase (BFC-LCC) algorithm (ζ = 1, η = 3) can achieve about 0.23 dB and 0.46 dB of signal-to-noise ratio (SNR) defined gain over the prior-art decoding algorithm at a frame error rate (FER) = $10^{-1}$. Compared with the ABP algorithm, the proposed CVNS-ABP algorithm and BFC-CVNS-ABP algorithm achieve performance gains of 0.18 dB and 0.23 dB, respectively, at FER = $10^{-3}$.

## 1. Introduction

Compared to separate source–channel coding (SSCC) schemes, joint source–channel coding (JSCC) can improve the coding gain of the channel decoder by exploiting the redundant information of the source [1]. The source redundancy can provide more effective protection for data transmission under the condition of resource-constrained. In addition, JSCC technology can significantly reduce end-to-end latency [2]. Therefore, it is suitable for the industrial Internet of Things (IoT) scenarios with limited resources, low error coding, and high real-time requirements [3,4,5].

The JSCC schemes based on low-density parity-check (LDPC) codes [1] uses a fixed-to-fixed length source encoder to compress the independent and identically distributed (i.i.d.) Bernoulli source and an LDPC code to protect the compressed source against channel errors. In [6,7], many works have been made in establishing the matching relationship between the basic modules in the transmission scheme based on double protograph LDPC (DP-LDPC) code and optimizing the JSCC system to obtain greater coding gain. The JSCC scheme of correlated sources based on systematic polar codes is proposed in [8], which uses the correlation between sources to improve the decoding performance. In [9], a distributed JSCC scheme based on polar codes is proposed, which can achieve high decoding performance without increased complexity. A joint source–channel (JSC) decoder based on belief propagation [10] is proposed to improve the efficiency of channel decoding when using the source information residual in the compressed bits. Although the JSCC scheme based on LDPC codes shows excellent decoding performance in wireless communication systems, it also has some shortcomings. The current source data is limited to i.i.d. Bernoulli source, and the code rate adjustment of LDPC codes is not flexible enough resulting in the en/decoder of each code rate not being universal. Recently, the source compression scheme based on RS codes is proved to have high speed [11] and coding flexibility [12]. Moreover, the JSCC scheme based on Reed–Solomon (RS) codes [13] can realize the efficient configuration of hardware resources and has high hardware efficiency. Although the JSCC scheme based on RS codes has the advantages of flexible parameters and outstanding hardware performance, there has been no relevant report on the efficiency and reliability of transmission. How to design an efficient JSC coding mode and apply the high-performance decoding algorithms of RS codes, such as the adaptive belief propagation (ABP) algorithm [14] and low-complexity chase (LCC) algorithm [15], to JSC decoding in order to further establish the relationship between source and channel decoding and realize high-performance decoding have become new challenges.

In coding theory, the bit flipping (BF) algorithm is normally used for the LDPC decoder proposed by Gallager [16], which is the simplest but most practical decoding algorithm for LDPC codes. In the last few years, BF has received renewed attention due to its usage in cryptosystems, in which one must guarantee a provably low failure rate [17,18]. This paper attempts to realize the BF of unreliable bits through source redundancy check and further realize the error detection and error correction of compressed source blocks. To date, a method to establish the connection between source and channel decoding with higher coding gain than the independent coding is missing, and little is known for further improving the efficiency of JSC coding. In this paper, we focus on the high-efficient encoding scheme and high-performance decoding algorithm for JSCC systems based on RS codes. The main contribution of this paper is summarized as follows:

(1) A novel joint coding matrix is constructed through which the source compression and channel coding can be realized simultaneously to improve the coding efficiency.
(2) A bit flipping check (BFC) algorithm is proposed to check and correct the errors of the compressed source block. The unreliable bits of each source block are selected by using the channel soft information to flip. If the reconstruction results of partial and all the source information are the same, the verification is successful.
(3) A controversial variable node selection-based ABP (CVNS-ABP) algorithm is presented for JSC decoding. The proposed CVNS-ABP algorithm reduces the influence of error bits on decoding by selecting error variable nodes (VNs) from controversial VNs and adding them to the sparsity of the parity-check matrix. Based on the BFC algorithm and CVNS-ABP algorithm, several JSC decoding algorithms are proposed. By making full use of the redundancy of the source, the performance of JSC decoding is greatly improved.

The rest of the paper is as follows. Section 2 introduces the JSC encoding using the proposed joint coding matrix and the JSC decoding schemes using the proposed BFC algorithm and CVNS-ABP algorithm. The simulation and comparison results are shown in Section 3. Section 4 draws the conclusion.

## 2. System Description

**Figure 1:**
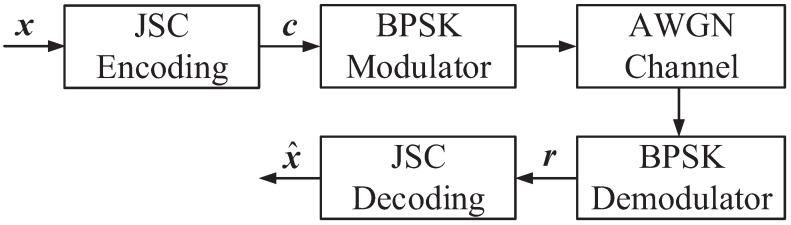
Block diagram of the JSCC system based on double RS codes.

The block diagram of the JSCC system in this paper is shown in Figure 1. The system is composed of double RS codes, in which the outer and the inner codes realize source compression and channel coding, respectively. The traditional JSC coding method [13] compresses the sparse source message x into a compressed source sequence s through the outer RS code source encoder, and then, it uses the inner RS code for channel coding. However, the two-step implementation of coding affects the efficiency of the system. For the sender, we propose a one-step joint coding method of source compression and channel coding. Binary phase-shift keying (BPSK) is used to modulate codeword c before transmission over the additive white Gaussian noise (AWGN) channel. At the receiver, the BFP algorithm and CVNS-ABP algorithm are proposed to achieve significant coding gain to accurately reconstruct the source message $x^{\prime }$.

### 2.1. JSC Encoding

For a *K*-degree sparse source, with most of its symbols being non-zero symbols, $x_{1}=\left(x_{1},x_{2},\ldots ,x_{N}\right),x_{i}\in GF\left(q=2^{m}\right)=\left\{0,1,\beta ,\beta^{2},\cdots ,\beta^{q-2}\right\}$, 1≤i≤N, where *K* represents the number of non-zero symbols, *N* represents the length of the source, the compressed source sequence $s_{1}=\left(s_{1},s_{2},\ldots ,s_{2K},\ldots ,s_{P}\right)\in GF\left(2^{m}\right)$ is computed by $s_{1}=H_{s}\cdot x_{1}^{T}$, where *P* is a little greater than 2K, for example, P=2K+2. This means that there are P−2K redundant symbols in $s_{1}$. $H_{s}$ is a source compression matrix over Galois field GF(q) with Vandermonde structure and is given by

(1) $$H_{s}=\left[\begin{array}{ccccc} 1 & \beta & \beta^{2} & \cdots & \beta^{N-1}\\ \vdots & \vdots & \vdots & \cdots & \vdots \\ 1 & \beta^{2K} & \beta^{4K} & \cdots & \beta^{(N-1)2K}\\ \vdots & \vdots & \vdots & \ddots & \vdots \\ 1 & \beta^{P} & \beta^{2P} & \cdots & \beta^{(N-1)P} \end{array}\right]$$

where P<N, β is a primitive element of $GF(2^{m})$.

The (n,k) RS code over finite field $GF\left(q\right)$ is used to complete the channel coding, which is constructed with the generator polynomial g(x) whose roots are 2t consecutive powers of β,

(2) $$\begin{array}{cc} g(z) & =\prod_{i=1}^{2t}\left(z-\beta^{i}\right)=\left(z-\beta \right)\left(z-\beta^{2}\right)\cdots \left(z-\beta^{2t}\right)\\ \; & =g_{0}+g_{1}z+g_{2}z^{2}\cdots g_{2t-1}z^{2t-1}+z^{2t} \end{array}$$

where n=k+2t is the code length, *k* is the number of information symbols, and 2t is the the number of parity symbols.

**Figure 2:**
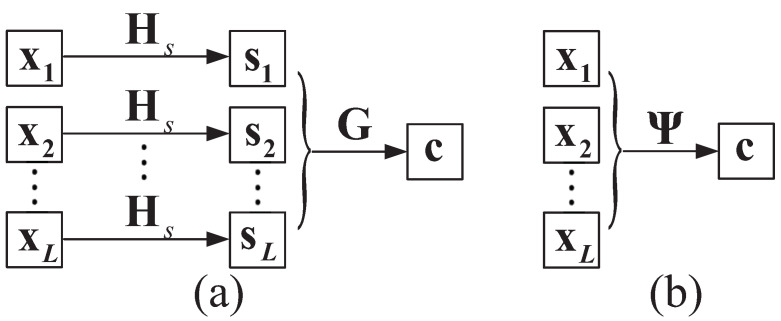
Schematic diagram of the coding process based on RS codes. (**a**) SSCC. (**b**) JSCC.

As described above and shown in Figure 2, the traditional SSCC scheme needs data compression first and then channel coding. However, in order to simplify the process of JSC coding, we construct a novel joint coding matrix, which can realize the source compression and channel coding of multiple strings of information simultaneously. Next, we will introduce the construction method of end-to-end JSC coding matrix. Firstly, the parity-check matrix $H_{c}$ of channel coding can be expressed as follows.    

(3) $$H_{c}=\left[\begin{array}{ccccc} 1 & \beta & \beta^{2} & \cdots & \beta^{n-1}\\ 1 & \beta^{2} & {\left(\beta^{2}\right)}^{2} & \cdots & {\left(\beta^{2}\right)}^{n-1}\\ 1 & \beta^{3} & {\left(\beta^{3}\right)}^{2} & \cdots & {\left(\beta^{3}\right)}^{n-1}\\ \vdots & \vdots & \vdots & \ddots & \vdots \\ 1 & \beta^{2t} & {\left(\beta^{2t}\right)}^{2} & \cdots & {\left(\beta^{2t}\right)}^{n-1} \end{array}\right]$$

Secondly, the matrix (3) can be transformed into a new matrix by the Gaussian elimination method as

(4) $$\begin{array}{cc} H_{c} & =\left[I_{2t}|Q\right]\\ \; & =\left[\begin{array}{ccccccccc} 1 & 0 & 0 & \cdots & 0 & b_{1,1} & b_{1,2} & \cdots & b_{1,k}\\ 0 & 1 & 0 & \cdots & 0 & b_{2,1} & b_{2,2} & \cdots & b_{2,k}\\ 0 & 0 & 1 & \cdots & 0 & b_{3,1} & b_{3,2} & \cdots & b_{3,k}\\ \vdots & \vdots & \vdots & \ddots & \vdots & \vdots & \vdots & \ddots & \vdots \\ 0 & 0 & 0 & \cdots & 1 & b_{2t,1} & b_{2t,2} & \cdots & b_{2t,k} \end{array}\right] \end{array}$$

The matrix (4) consists of 2t×2t identity matrix $I_{2t}$ and a Q matrix, where the elements of the Q matrix are the elements on the finite field $GF(q=2^{m})$ obtained by matrix transformation. Then, the generation matrix $G_{c}$ of the RS code can be constructed through matrix Q and a k×k identity matrix $I_{k}$ as

(5) $$\begin{array}{cc} G_{c} & =\left[Q^{T}|I_{k}\right]\\ \; & =\left[\begin{array}{ccccccccc} b_{1,1} & b_{2,1} & \cdots & b_{2t,1} & 1 & 0 & 0 & \cdots & 0\\ b_{1,2} & b_{2,2} & \cdots & b_{2t,2} & 0 & 1 & 0 & \cdots & 0\\ b_{1,3} & b_{2,3} & \cdots & b_{2t,3} & 0 & 0 & 1 & \cdots & 0\\ \vdots & \vdots & \ddots & \vdots & \vdots & \vdots & \vdots & \ddots & \vdots \\ b_{1,k} & b_{2,k} & \cdots & b_{2t,k} & 0 & 0 & 0 & \cdots & 1 \end{array}\right] \end{array}$$

To make the compressed information dimension consistent with the information dimension in channel coding, a novel source compression matrix $\Phi_{LP\times LN}$ can be constructed through the observation matrix $H_{s}$ (1) as

(6) $$\Phi_{LP\times LN}=\left[\begin{array}{ccccc} H_{s} & 0 & 0 & \cdots & 0\\ 0 & H_{s} & 0 & \cdots & 0\\ 0 & 0 & H_{s} & \cdots & 0\\ \vdots & \vdots & \vdots & \ddots & \vdots \\ 0 & 0 & 0 & \cdots & H_{s} \end{array}\right]$$

where *L* represents the number of $H_{s}$ matrices.

Finally, the proposed JSC coding matrix Ψ, where k=LP, can be constructed as

(7) $$\Psi =G_{c}^{T}\cdot \Phi_{LP\times LN}$$

A plurality of information blocks $x_{1}$, $x_{2}$, $x_{3}$,⋯, $x_{L}$ are constructed into a long information block x as

(8) $$\begin{array}{cc} x & ={\left(x_{1},x_{2},\cdots x_{L}\right)}^{T}\\ \; & ={\left(x_{1,1},x_{1,2},\cdots x_{1,N},\cdots ,x_{L,1},x_{L,2},\cdots ,x_{L,N}\right)}^{T} \end{array}$$

Through the constructed JSC matrix Ψ, the JSC of long information blocks can be completed as the following:

(9) c=Ψ·x.

### 2.2. Bit Flipping Check Algorithm

Compared with the traditional JSC decoding [13], the proposed scheme needs to check and correct the errors of each compressed source block first. As shown in Algorithm 1, the log-likelihood ratios (LLRs) (10) of each received compressed source bit ${\hat{s}}_{i,j}$ are calculated and the ζ least reliable bits whose LLRs approach 0 are selected in step 1, where $\sigma^{2}$ is the variance of noise vector.

(10) $$\Gamma_{i,j}=\ln\frac{p\left\{s_{i,j}^{\prime }=1|{\hat{s}}_{i,j}\right\}}{p\left\{s_{i,j}^{\prime }=0|{\hat{s}}_{i,j}\right\}}=\frac{2{\hat{s}}_{i,j}}{\sigma^{2}}$$

**Algorithm 1:** The Bit Flipping Check Algorithm

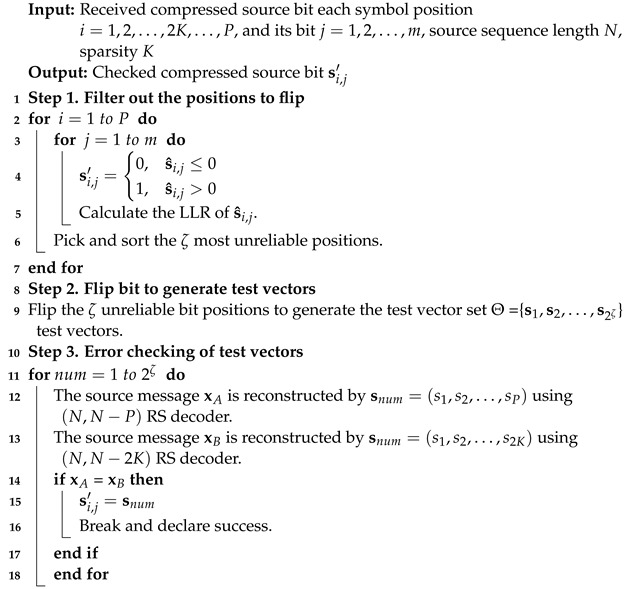



In step 2, the selected ζ unreliable bits are flipped to obtain $2^{\zeta }$ test vectors for checking and decoding. The larger the ζ value, the more source test vectors are selected, and the better the decoding performance. For the correct source test vector, the compressed source symbol $s_{i}$(i=1,2,…,2K,…,P), source sequence location χ, and value δ satisfy the following equation:

(11) $$\begin{array}{c} s_{i}=\delta_{1}\chi_{1}^{i}+\delta_{2}\chi_{2}^{i}+\delta_{3}\chi_{3}^{i}+\cdots +\delta_{K}\chi_{K}^{i} \end{array}$$

Obviously, due to the redundancy of compressed source symbols, only 2K compressed source symbols are needed to reconstruct the source sequence with sparsity *K*. Assume that the source sequence has *K* non-zero symbols in positions $o_{1},o_{2},\ldots ,o_{K}$. The polynomial of source sequence positions is defined as

(12) $$\xi \left(z\right)=\prod_{j=1}^{K}\left(1-\chi_{j}z\right)=p_{0}+p_{1}z+p_{2}z^{2}+\cdots +p_{K}z^{K}$$

where $\beta_{j}=\alpha^{o_{j}}$ are related to the target symbol locations. The polynomial of source sequence values is defined as

(13) $$\begin{array}{cc} \upsilon (z) & =\sum_{j=1}^{K}u_{j}\chi_{j}\prod_{i=1,i\neq j}^{K}\left(1-\chi_{i}z\right)\\ \; & =q_{0}+q_{1}z+q_{2}z^{2}+\cdots +q_{K-1}z^{K-1} \end{array}$$

where $u_{j}$ represents the target symbol values.

We can get the following parameter equation

(14) $$\xi \left(z\right)s\left(z\right)=\upsilon \left(z\right)\;\;\mathrm{mod}\;z^{2K}$$

which can be solved by the reformulated inversionless Berlekamp–Massey (RiBM) algorithm [19], and then, the source information can be reconstructed by the Forney algorithm.

(15) $$x_{A}=v\left(\chi_{j}^{-1}\right)/\xi^{\prime }\left(\chi_{j}^{-1}\right)$$

Similarly, the source information $x_{B}$ can be reconstructed by using all the *P* compressed source symbols. If there is no error in the compressed source block, the reconstruction results using the first 2K symbols and *P* symbols should be the same. Therefore, we can use this method to verify whether there are errors in the received source sequence. If the reconstructed source message $x_{A}$ and $x_{B}$ are the same, it means that the test vector is the correct source sequence. Moreover, the more unreliable bit positions are selected, the greater the probability of successful source error correction.

### 2.3. Controversial Variable Node Selection Based Adaptive Belief Propagation Decoding Algorithm

In order to further improve the performance of the JSC decoder, it is necessary to introduce the ABP algorithm to reduce or eliminate error bits. The ABP algorithm uses the idea of hierarchical statistics to adjust the parity-check matrix to avoid error transmission in the process of BP decoding. According to the LLRs, some unreliable VNs with small LLRs can be determined. However, there are some controversial nodes with small LLRs between unreliable VNs and reliable VNs. Since their LLRs have a slight difference to determine whether they are reliable, they are called controversial VNs. If they are only distinguished according to the little LLR difference, some error VNs may be determined as reliable VNs, resulting in the wide-range transmission of error information to many check nodes (CNs).

**Figure 3:**
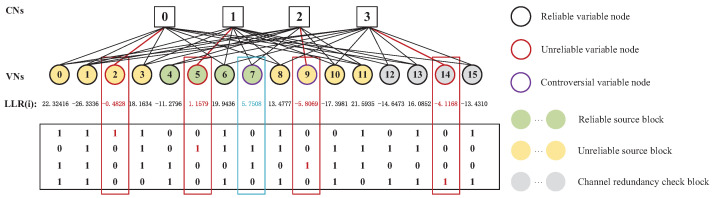
Illustration of the proposed CVNS-ABP decoding scheme.

The proposed CVNS-ABP algorithm in Algorithm 2 improves the performance of the ABP algorithm in joint decoding by selecting VNs with high error probability among controversial VNs. As shown in Figure 3, firstly, we select the ρ unreliable VNs with the smallest absolute LLRs. For some VNs whose unreliability is second only to the selected ρ unreliable VNs, their unreliability has little difference, which is called controversial VNs. If θ sub unreliable nodes are selected from the controversial VNs only according to their slight unreliability difference, some possible error nodes will degrade the decoding performance. The unreliable source block is selected by the BFC algorithm, and then, θ unreliable VNs are selected in the unreliable source block. With the increase of signal-to-noise ratio (SNR), the controversial VNs will decrease, and the value of θ should be reduced accordingly to obtain good performance. Our proposed CVNS-ABP algorithm reduces the propagation of error information and improves the performance of iterative decoding by accurately selecting unreliable VNs and controversial VNs that may have errors and comparing them with the corresponding parity-check matrix columns.    
**Algorithm 2:** The CVNS-ABP Algorithm**Initialize:** Set damping factor α, the maximum iteration number $i_{max}$, the LLR of the *i*-th iteration $L^{\left(i\right)}$.**Step 1. BFC for each source block**Calculate the $x_{A}$ and $x_{B}$ for each source block.**if***$x_{A}$ = $x_{B}$***then**(    

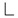
 Declare the checked source block is reliable.**else**(     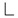 Declare the checked source block is unreliable.**Step 2. Unreliable VN selection**Sort the bit sequence according to $L^{\left(i\right)}$ and select ρ unreliable VNs with the smallest absolute value.**Step 3. Controversial VN selection**Select the remaining θ controversial VNs from the unreliable source blocks, where ρ + θ = m×(n−k).**Step 4. Parity-check matrix update**Implement Gaussian elimination to unitize the ρ+θ unreliable positions selected in the parity-check matrix.**Step 5. Extrinsic information generation**Calculate the extrinsic LLR vector $L_{\mathrm{ext}}^{\left(i\right)}$ $L_{\mathrm{ext}}^{\left(i\right)}\left(v_{n}\right)=\sum_{c_{m}\in M\left(v_{n}\right)}2\tanh^{-1}$
$$\left(\prod_{v_{j}\in N\left(c_{m}\right)\setminus v_{n}}\tanh\left(\frac{L^{\left(i\right)}\left(v_{j}\right)}{2}\right)\right)$$
where $v_{n}$ is the *n*-th coded bit of the sequence c and $c_{m}$ is the *m*-th check node, $M\left(v_{n}\right)$ and $N\left(c_{m}\right)$ denote the set of $v_{n}$ and $c_{m}$, respectively. $\;\;\;N\left(c_{m}\right)\backslash v_{n}$ denotes the subset $M\left(v_{n}\right)$ without $v_{n}$.**Step 6. Bit-level reliabilities update**$L^{\left(i+1\right)}=L^{\left(i\right)}+\alpha L_{ext}^{\left(i\right)}$, where 0<α≤1.**Step 7. Hard decision**Make a hard decision on the value of VNs ${\hat{c}}_{j}=\left\{\begin{array}{cc} 0, & L^{\left(i+1\right)}\left(c_{j}\right)>0\\ 1, & L^{\left(i+1\right)}\left(c_{j}\right)<0 \end{array}\right.$**Step 8. Termination criterion****if***$S_{j}\;=\;0,$j=1,2,⋯,2t***then**(     

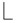
 output the estimated bits.**else if**$i=i_{max}$**then**(    

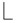
 output the estimated bits.**else**(    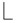 return to **Step 1**.

### 2.4. High-Performance JSC Decoding Scheme

**Figure 4:**
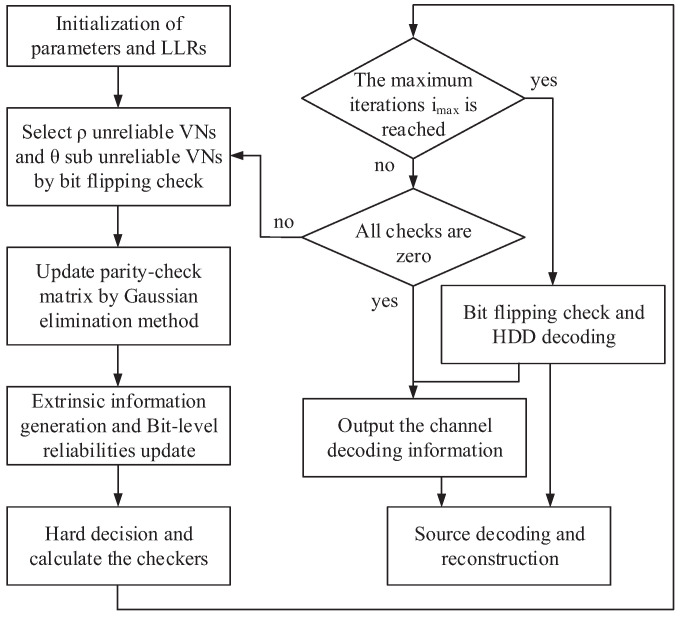
Schematic diagram of the proposed BFC-CVNS-ABP algorithm.

The main advantage of JSCC is to apply the redundancy of the compressed source sequence to improve the coding gain of channel decoding. However, the previous JSCC schemes [13] for RS codes pay more attention to the integration of decoders, and there are no relevant reports on the scheme of improving channel coding gain. The proposed BFC algorithm and CVNS-ABP algorithm provide high-performance decoding methods for the JSCC scheme based on double RS codes. A novel high-performance JSC decoding algorithm called BFC-CVNS-ABP is obtained by combining the two algorithms, and its flow chart is shown in Figure 4. The proposed JSC decoding scheme reduces or eliminates the error bits through the CVNS-ABP algorithm, and it outputs the decoding result when the parity checks are all zero. Otherwise, when the maximum number of iterations is reached, the BFC algorithm is performed to flip and correct some errors. Each source block is verified by the BFC algorithm. If the verification is successful, the reconstructed signal $x_{1}$ is output. Then, we execute hard-decision decoding (HDD) to solve the remaining errors. After successful channel decoding, the BM algorithm is used to reconstruct the source information. Finally, we select the compressed source block that cannot be successfully decoded by the BFC algorithm for reconstruction.

## 3. Simulation Results and Discussion

**Figure 5:**
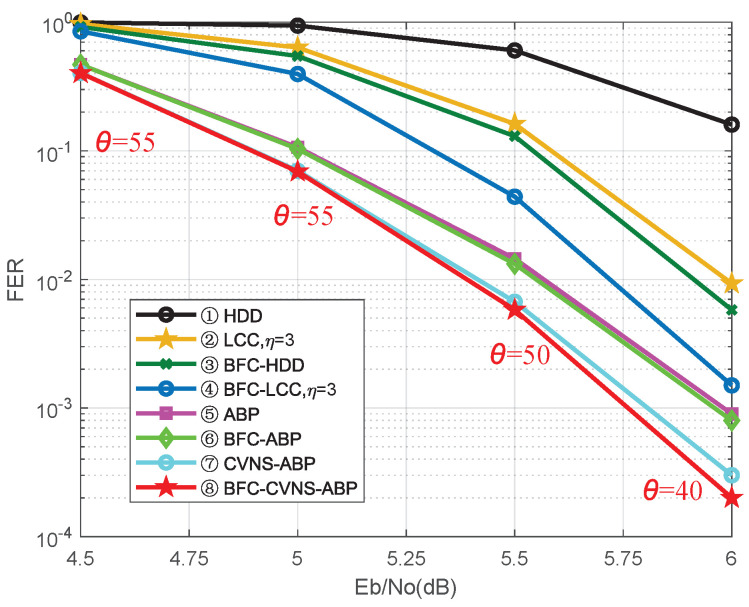
Frame error rate (FER) versus Eb/No of the proposed JSC decoding algorithms compared with other algorithms.

In this section, we illustrate the advantages of the proposed JSC algorithms through simulation. We combine the proposed BFC algorithm and CVNS-ABP algorithm with other algorithms to obtain several novel JSC decoding algorithms for comparison. The JSC coding parameters are *q* = 256, *m* = 8, *K* = 7, *P* = 16, *L* = 14, *n* = 240, *k* = 224. The encoded codewords are transmitted in the AWGN channel. In Figure 5, we compare the frame error rate (FER) performances of the three previous algorithms with the five proposed algorithms. As shown in Figure 5, the proposed BFC-HDD algorithm (ζ = 1) and BFC-LCC algorithm (ζ = 1, η = 3) can achieve about 0.55 dB and 0.78 dB of SNR gain over the decoding scheme in [13] at FER = $10^{-1}$, where η is the selected unreliable positions of the channel RS code. In order to further improve the performance of JSC decoding, the ABP algorithm is improved. The combination of the proposed BFC algorithm and ABP algorithm has a little performance improvement. Compared with the ABP algorithm [14], with a maximum iteration number of $i_{max}$ = 60, the proposed CVNS-ABP algorithm and BFC-CVNS-ABP algorithm achieve performance gains of 0.18 dB and 0.23 dB, respectively, at FER = $10^{-3}$.

**Figure 6:**
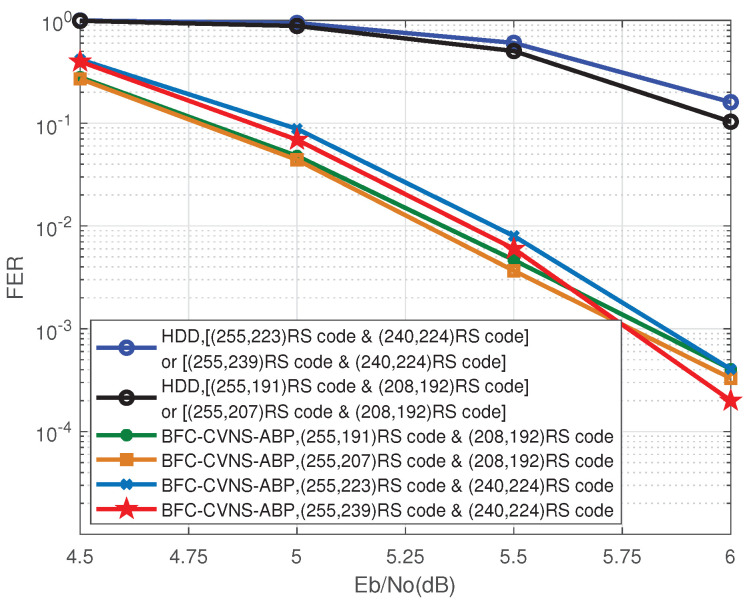
Frame error rate (FER) versus Eb/No of the proposed JSC decoding algorithm for different source sparsity.

Figure 6 shows the JSC decoding performance of the proposed BFC-CVNS-ABP algorithm under different source sparsity: (255, 239) RS code, (255, 223) RS code, (255, 207) RS code, and (255, 191) RS code are selected to encode sparse sources with the length *N* of 255 and the sparsity *K* of 7, 15, 23, and 31, respectively. Meanwhile, (208, 192) RS code and (240, 224) RS code are selected for channel coding. In the same channel coding mode, the smaller the sparsity of the source, the more accurate the controversial VNs selected by the BFC algorithm, and the better the performance of JSC decoding.

## 4. Conclusions

This paper proposes a novel JSCC scheme based on double RS codes. We construct a new JSC coding matrix, which can synchronously realize source compression and channel decoding to reduce system latency. In order to improve the coding gain of JSC decoding, the BFC algorithm and CVNS-ABP algorithm are proposed, where the BFC algorithm can check and correct the source error bits by making full use of a little source redundancy, and the CVNS-ABP algorithm realizes high-performance decoding by selecting the controversial VNs of the error source block and sparsifying the corresponding parity-check matrix columns. Simulation results show that the JSC decoding algorithm based on the BFC algorithm and CVNS-ABP algorithm can significantly improve the coding gain. Future work will focus on exploring the implementation of JSC decoding based on LDPC codes for sparse sources.

## Data Availability

Not applicable.

## References

[1] Fresia M., Perez-Cruz F., Poor H.V., Verdu S. (2010). Joint source and channel coding. IEEE Signal Process. Mag..

[2] Zhong Y., Alajaji F., Campbell L.L. (2006). On the joint source–channel coding error exponent for discrete memoryless systems. IEEE Trans. Inf. Theory.

[3] Chen X. (2018). Zero-delay Gaussian joint source–channel coding for the interference channel. IEEE Commun. Lett..

[4] Fresnedo O., Suarez-Casal P., Castedo L. (2019). Transmission of spatiotemporal correlated sources over fading multiple access channels with DQLC mappings. IEEE Trans. Commun..

[5] Deng L., Shi Z., Li O., Ji J. (2019). Joint Coding and Adaptive Image Transmission Scheme Based on DP-LDPC Codes for IoT Scenarios. IEEE Access.

[6] Chen Q., Wang L. (2020). Design and analysis of joint source channel coding schemes over non-standard coding channels. IEEE Trans. Veh. Technol..

[7] Liu S., Wang L., Chen J., Hong S. (2020). Joint Component Design for the JSCC System Based on DP-LDPC Codes. IEEE Trans. Commun..

[8] Wang Y., Qin M., Narayanan K.R., Jiang A., Bandic Z. Joint source-channel decoding of polar codes for language-based sources. Proceedings of the 2016 IEEE Global Communications Conference (GLOBECOM).

[9] Jin L., Yang P., Yang H. (2018). Distributed Joint Source-Channel Decoding Using Systematic Polar Codes. IEEE Commun. Lett..

[10] Dong Y., Niu K., Dai J., Wang S., Yuan Y. (2021). Joint Source and Channel Coding Using Double Polar Codes. IEEE Commun. Lett..

[11] Wang H., Zhang W., Liang Y., Liu Y. (2020). Efficient Reconstruction Architecture of Compressed Sensing and Integrated Source-Channel Decoder Based on Reed Solomon Code. IEEE Commun. Lett..

[12] Wang H., Zhang W., An X., Liu Y. (2020). Sparsity Adaptive Compressed Sensing and Reconstruction Architecture Based on Reed-Solomon Codes. IEEE Commun. Lett..

[13] Wang H., Zhang W., Liu Y. Joint Coding Scheme Based on Reed-Solomon Codes. Proceedings of the 2021 IEEE 6th International Conference on Computer and Communication Systems (ICCCS).

[14] Jiang J., Narayanan K.R. (2006). Iterative soft-input soft-output decoding of Reed-Solomon codes by adapting the parity-check matrix. IEEE Trans. Inf. Theory.

[15] Valls J., Torres V., Canet M.J., García-Herrero F.M. (2019). A test vector generation method based on symbol error probabilities for low complexity chase soft-decision Reed-Solomon decoding. IEEE Trans. Circ. Syst. I Reg. Pap..

[16] Gallager R. (1962). Low-density parity-check codes. IEEE Trans. Inf. Theory.

[17] Santini P., Battaglioni M., Baldi M., Chiaraluce F. (2020). Analysis of the error correction capability of LDPC and MDPC codes under parallel bit-flipping decoding and application to cryptography. IEEE Trans. Commun..

[18] Tillich J.-P. The decoding failure probability of MDPC codes. Proceedings of the 2018 IEEE International Symposium on Information Theory (ISIT).

[19] Sarwate D.V., Shanbhag N.R. (2001). High-speed architectures for Reed-Solomon decoders. IEEE Trans. Large Scale Integr. (VLSI) Syst..

